# Relationship between ABO blood groups and gestational hypertensive disorders: A protocol for systematic review and meta-analysis

**DOI:** 10.1097/MD.0000000000025573

**Published:** 2021-05-07

**Authors:** Nuerbiye Dilixiati, Shuang Sui, Xinmei Ge, Dilihuma Tuerxun, Ying Huang

**Affiliations:** People's Hospital of Xinjiang Uygur Autonomous Region, Urumqi 830001, Xinjiang Uygur Autonomous Region Province, China.

**Keywords:** ABO blood group, gestational hypertensive disorders, meta-analysis, protocol

## Abstract

**Background:**

The distribution of ABO blood group is related to the incidence of various diseases. Gestational hypertensive disorders (GHD) is one of the most important risk factors during pregnancy, which has certain heredity. It is reported that ABO blood type is associated with the risk of GHD. However, the results are still controversial. In this study, we conducted a systematic review and meta-analysis to clarify the relationship between ABO blood group and GHD.

**Methods:**

All eligible studies come from Embase, Cochrane Library, Pubmed, Chinese databases SinoMed, Chinese National Knowledge Infrastructure, Chinese Scientific Journals Database, and Wanfang Data. The retrieval time is from the establishment of the database to March 2021. The language will be limited to Chinese and English. The 2 reviewers will be responsible for the selection of the study, the extraction of data, and the evaluation of the quality of the research. Pooled odds ratios (ORs) with 95% confidence intervals (CIs) were used to assess the corresponding associations. Sensitivity analysis, publication bias assessment, and heterogeneity test were performed using STATA 16.0.

**Results:**

The results of this meta-analysis will be published in peer-reviewed journals.

**Conclusion:**

This study will provide evidence to support the relationship between ABO blood group and the risk of GHD.

**Ethics and dissemination:**

The private information from individuals will not be published. This systematic review also will not impair endangering participants’ rights. Ethical approval is not required. The results may be published in a peer-reviewed journal or disseminated in relevant conferences.

**OSF Registration number:**

DOI 10.17605/OSF.IO/3X9YZ.

## 1 Introduction

Gestational hypertensive disorder (GHD) is a common pregnancy-specific disease, which can lead to varying degrees of maternal and fetal injury, and even endanger the safety of life.^[1,2]^ The disease usually occurs after 20 weeks of gestation and is characterized by hypertension, albuminuria, and other systemic dysfunctions. Together with postpartum hemorrhage, complications of pregnancy with heart disease and puerperal infections, it constitutes 1 of the 4 major causes of maternal death. In China, the incidence of GHD is about 9.4%,^[3]^ while it is reported that the incidence of GHD is 16% abroad.^[4]^

Up to now, the etiology of GHD is not very clear. Over the years, researchers have put forward many theories, including uterine and placental local ischemia, vascular endothelial injury, immune factors, and genetic factors.^[4]^

Blood type is a very stable genetic trait, which is inherited by autosomal dominant or recessive mode. Kumar et al believe that human ABO blood group is the result of evolutionary selection mediated by environment and pathogens, and that there is some inevitable relationship between ABO blood group and disease.^[5]^ Current studies have found that ABO blood group is associated with tumor, hepatitis B, *Helicobacter pylori* infection, coronary artery disease, and other diseases.^[6–10]^

At present, the research conclusions about the influence of ABO blood group system on the occurrence and development of GHD are very contradictory. Many reports show that blood type has obvious or no obvious influence on the occurrence of GHD.^[11–18]^ Therefore, the purpose of this study is to explore the effect of blood type on the risk of GHD by meta-analysis, and to provide a basis for clinical evaluation of GHD.

## 2 Methods

### 2.1 Study registration

The protocol was registered in Open Science Framework (registration number: DOI 10.17605/OSF.IO/3X9YZ). This systematic review and meta-analysis will be reported inconformity with the preferred reporting items for systematic reviews and meta-analysis protocols (PRISMA-P) 2015.^[19]^

### 2.2 Ethic

The review does not involve the assessment of patients’ individual information or rights, so there is no need to obtain approval from the ethical institution.

### 2.3 Inclusion criteria

Studies would be included in this meta-analysis based on the following criteria:

1) case–control study, cohort study, and cross-sectional study will be included;
2) study related to ABO blood type and GHD susceptibility;
3) any GHD during the pregnancy;
4) the relationship between ABO blood type and GHD provides odds ratio (OR) and 95% confidence interval (95% CI).

### 2.4 Exclusion criteria

1) Repeated published studies;
2) literature with missing or unusable data;
3) reviews, meta-analyses, case series.

### 2.5 Search strategy

Data was searched in Embase, Cochrane Library, Pubmed, Chinese databases SinoMed, Chinese National Knowledge Infrastructure, Chinese Scientific Journals Database (VIP), and Wanfang Data. The retrieval time is from the establishment of the database to March 2021. The language will be limited to Chinese and English. The details of PubMed's search strategy are shown in Table 1, including all search terms, while similar search strategies are applied to other electronic databases.

**Table 1:** Search strategy in PubMed database.

Number	Search terms
#1	Gestational hypertensive disorders[Title/Abstract]
#2	Pregnancy-induced hypertension[Title/Abstract]
#3	Gestational hypertension[Title/Abstract]
#4	Hypertensive disorder complicating pregnancy [Title/Abstract]
#5	Hypertension in pregnancy[Title/Abstract]
#6	Gestineing hypertension[Title/Abstract]
#7	or/1–6
#8	ABO Blood-Group System[MeSH]
#9	ABH Blood Group[Title/Abstract]
#10	ABO Factors[Title/Abstract]
#11	Blood Group H Type 1 Antigen[Title/Abstract]
#12	H Blood Group[Title/Abstract]
#13	H Blood Group System[Title/Abstract]
#14	ABH Blood Groups[Title/Abstract]
#15	ABO Blood Group System[Title/Abstract]
#16	ABO Blood-Group Systems[Title/Abstract]
#17	ABO Factor[Title/Abstract]
#18	Blood Group, ABH[Title/Abstract]
#19	Blood Group, H[Title/Abstract]
#20	Blood Groups, ABH[Title/Abstract]
#21	Blood Groups, H[Title/Abstract]
#22	Blood-Group System, ABO[Title/Abstract]
#23	Blood-Group Systems, ABO[Title/Abstract]
#24	Factor, ABO[Title/Abstract]
#25	Factors, ABO[Title/Abstract]
#26	H Blood Groups[Title/Abstract]
#27	System, ABO Blood-Group[Title/Abstract]
#28	Systems, ABO Blood-Group[Title/Abstract]
#29	or/8–29
#30	#7 and #29

### 2.6 Data collection and analysis

#### 2.6.1 Selection of studies

**Figure 1:**
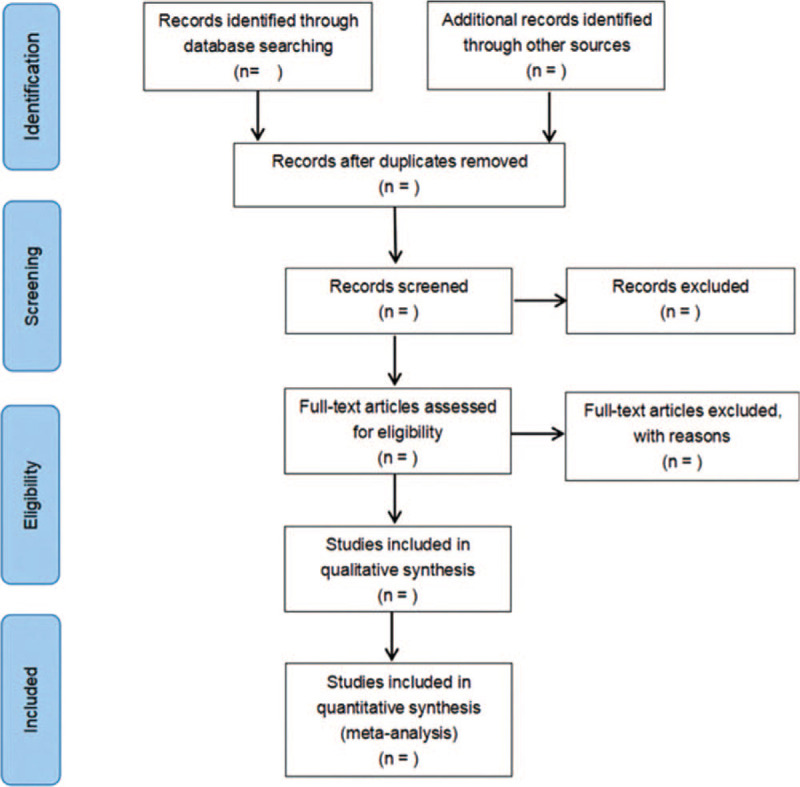
Flow diagram of study selection process.

The screening flow chart of this study is displayed in Figure 1. The literature and data were independently screened and crosschecked by 2 researchers. If there are differences to be discussed and resolved, if necessary, they would be submitted to a third researcher for adjudication.

#### 2.6.2 Data extraction

According to the piloted forms, the information was extracted independently by 2 reviewers from the selected studies: first author, year of publication, country, race of each study population, number of cases and controls, average age, ABO blood type, number of cases of GHD, etc.

#### 2.6.3 Study quality assessment

The quality of selected cohort studies was assessed using the Newcastle-Ottawa Scales (NOS).^[20,21]^ The quality of the selected cross-sectional studies was assessed using an 11-item checklist recommended by the Agency for Healthcare Research and Quality (AHRQ).^[22]^

#### 2.6.4 Dealing with missing data

In terms of studying the deficiencies of the raw data, we contacted the author by email and asked for the original data. If the original data is not available, then we would analyze the existing data.

### 2.7 Statistical analysis

The main outcome was the prevalence of GHD in our meta-analysis. A meta-analysis was conducted using STATA 16.0. The relationship between the ABO blood groups and prevalence of GHD was quantified using OR values and the corresponding 95% CIs. Between-study heterogeneity was evaluated with the *I*^2^ statistic. When *I*^2^ ≤ 50%, the included studies were considered to have little heterogeneity; when *I*^2^ > 50%, the included studies were considered to have substantial heterogeneity. *I*^2^ < 50% revealed that the studies exhibited homogeneity, then fixed effects model would be used. Otherwise, the random effects model would be adopted.

### 2.8 Subgroup analysis

According to study type, ethnicity, sample size, and age, we carried out subgroup analysis.

### 2.9 Sensitivity analysis

Sensitivity analysis used different combined effect models.

### 2.10 Assessment of publication biases

If more than 10 studies are included, a funnel chart would be applied to assess the report bias.^[23,24]^

## 3 Discussion

In recent years, through the analysis and study of clinical cases, many scholars have found that the distribution of ABO blood group is related to the incidence of various diseases. Recent genome-wide association studies have shown that genetic variation at the ABO locus is associated with soluble E-selectin, P-selectin, intercellular cell adhesion molecule-1, vascular inflammatory agents, which are associated with hypertension and type 2 diabetes.^[25–28]^ Lee et al show that women with AB blood group had the highest risk of developing GHD, while women with O blood group had the lowest risk.^[29]^ Rajakeerthana's study shows that the risk of PIH in pregnant women with non-O blood group is higher than that in pregnant women with O blood group.^[30]^ However, other studies have shown that ABO blood type is not associated with the risk of GHD.^[31]^ The purpose of this study is to systematically evaluate the risk relationship between ABO blood group and GHD in order to provide evidence-based medicine for clinical guidance in the future.

This meta-analysis analyzes the incidence and regularity of GHD from the perspective of genetics by analyzing the correlation between global GHD and ABO blood groups. This study is less involved in this field, and has a certain degree of innovation. At the same time, it is of great practical and theoretical significance for the prevention and treatment of GHD in the future.

## Author contributions

**Data collection:** Nuerbiye Dilixiati and Shuang Sui.

**Funding acquisition:** Shuang Sui, Ying Huang.

**Funding support:** Ying Huang and Shuang Sui.

**Investigation:** Xinmei Ge, Ying Huang.

**Literature retrieval:** Ying Huang and Xinmei Ge.

**Software operating:** Shuang Sui and Dilihuma Tuerxun.

**Software:** Shuang Sui, Dilihuma Tuerxun.

**Supervision:** Nuerbiye Dilixiati and Dilihuma Tuerxun.

**Writing – original draft:** Nuerbiye Dilixiati and Shuang Sui.

**Writing – review & editing:** Nuerbiye Dilixiati and Shuang Sui and Ying Huang.
